# Patient‐controlled subcutaneous analgesia using sufentainil or morphine in home care treatment in patients with stage III‐IV cancer: A multi‐center randomized controlled clinical trial

**DOI:** 10.1002/cam4.3194

**Published:** 2020-06-04

**Authors:** Cheng‐Fu Wan, Qing‐Zhu Meng, Yan‐Wei Wang, Liang Qi, Chang‐Liang Ai, Xin Sui, Tao Song

**Affiliations:** ^1^ Pain Department of the First Affiliated Hospital China Medical University Shenyang China; ^2^ Pain Department of the Central Hospital of Haicheng city Anshan China; ^3^ Pain Department of the Third People's Hospital of Anshan city Anshan China; ^4^ Pain Department of the Central Hospital of Fuxin city Fuxin China; ^5^ Pain Department of the Women and Children's Hospital of Dandong city Dandong China; ^6^ Pain Department of the Central Hospital of Kuandian city Dandong China

**Keywords:** 36‐item Short Form health survey, advanced cancer, numeric rating scale, patient‐controlled subcutaneous analgesia, sufentanil

## Abstract

**Purpose:**

Patient‐controlled subcutaneous analgesia (PCSA) with sufentanil is an alternative analgesia strategy in patients with stage III‐IV cancer; however, its efficacy and safety have not been fully investigated.

**Methods:**

From May 10, 2017 to November 10, 2017, 120 patients with stage III‐IV cancer suffering from moderate to severe pain were prospectively enrolled from six hospitals and randomized to receive PCSA with morphine (control group) or sufentanil (intervention group). Before the PCSA and on days 1, 3, 7, 14, 28, and 56 after treatment, the numeric rating scale (NRS) and 36‐item Short Form health survey (SF‐36) were completed for each patient and the side effects were also recorded.

**RESULTS:**

No significant differences (*P* > .05) were observed in the preoperative NRS score and the SF‐36 parameters between the two groups. Patients in the intervention group achieved better pain relief, as indicated by lower NRS scores at days 14 (*P* = .040), 28 (*P* < .001), and 56 (*P* < .001) after PCSA device implantation (vs control group). Furthermore, the patients in the intervention group also achieved a better life quality, as indicated by the physical role, general health, social function body pain, and mental health scores. Finally, the patients receiving sufentanil showed lower levels of nausea and somnolence than those in the control group.

**Conclusion:**

PCSA with sufentanil achieves better pain control and life quality as well as fewer adverse reactions in stage III‐IV cancer patients with pain and may be a promising pain management in these patients.

**Trial registration:**

This study was registered at chictr.org.cn with the trial number: ChiCTR‐IPR‐17011280.

## Graphical abstract

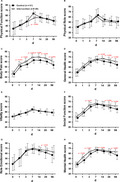

## 1 INTRODUCTION

Cancer is frequently associated with the disturbing symptom of pain. Currently, the worldwide pain prevalence is about 50% in cancer patients at the time of diagnosis and approximately 80% in patients with stage III‐IV cancers.^1^ Administration of opioid analgesics through varied routes is regarded as the cornerstone for cancer‐pain management. Oral administration is recommended by the World Health Organization.^2^ However, parenteral administration is also required, especially when patients cannot tolerate oral agents or need rapid‐onset analgesia.^3^

In the clinical setting, rapid‐onset analgesia can be achieved through a variety of methods, including subcutaneous (s.c.) or intravenous (iv) patient‐controlled analgesia (PCA),^4^ which are the major parenteral delivery techniques.^5^ PCA allows a continuous drug infusion via a programmable pump, providing a constant plasma concentration of analgesics, as well as enabling the patient to voluntarily control pain using on‐demand supplemental boluses.^6^ Furthermore, PCA enables the individual‐based analgesic titration to be more adaptable.^7^ All these features make PCA an ideal strategy in palliative care, outpatient care, and breakthrough cancer pain management.

Sufentanil, a highly lipophilic opioid fentanyl analogue, is typically used for surgical analgesia, especially in patients with renal impairment, based on pharmacokinetics and clinical experience.^8^ In the clinical setting, this drug is commonly iv applied to achieve rapid‐onset analgesia for cancer pain with less opioid tolerance.^9^ Although the injectable form of sufentanil is available in several countries ^10^ and subcutaneous (s.c.) administration is a simple and safe way for advanced cancer patients, the efficacy of continuous s.c. sufentanil infiltration has not been reported, except for a single‐center small cohort study.^11^ The most recent study investigating the efficacy of PCA sufentanil analgesia in cancer pain patients was a retrospective one for iv technique, not a prospective design involving the s.c. technique.^12^

Thus, the primary purpose of the current study is to prospectively evaluate the efficacy of patient‐controlled subcutaneous analgesia (PCSA) with sufentanil for home care stage III‐IV cancer patients. The secondary purpose is to evaluate the quality of life in these advanced cancer patients.

## 2 MATERIALS AND METHODS

### 2.1 Study participants

**FIGURE 1:**
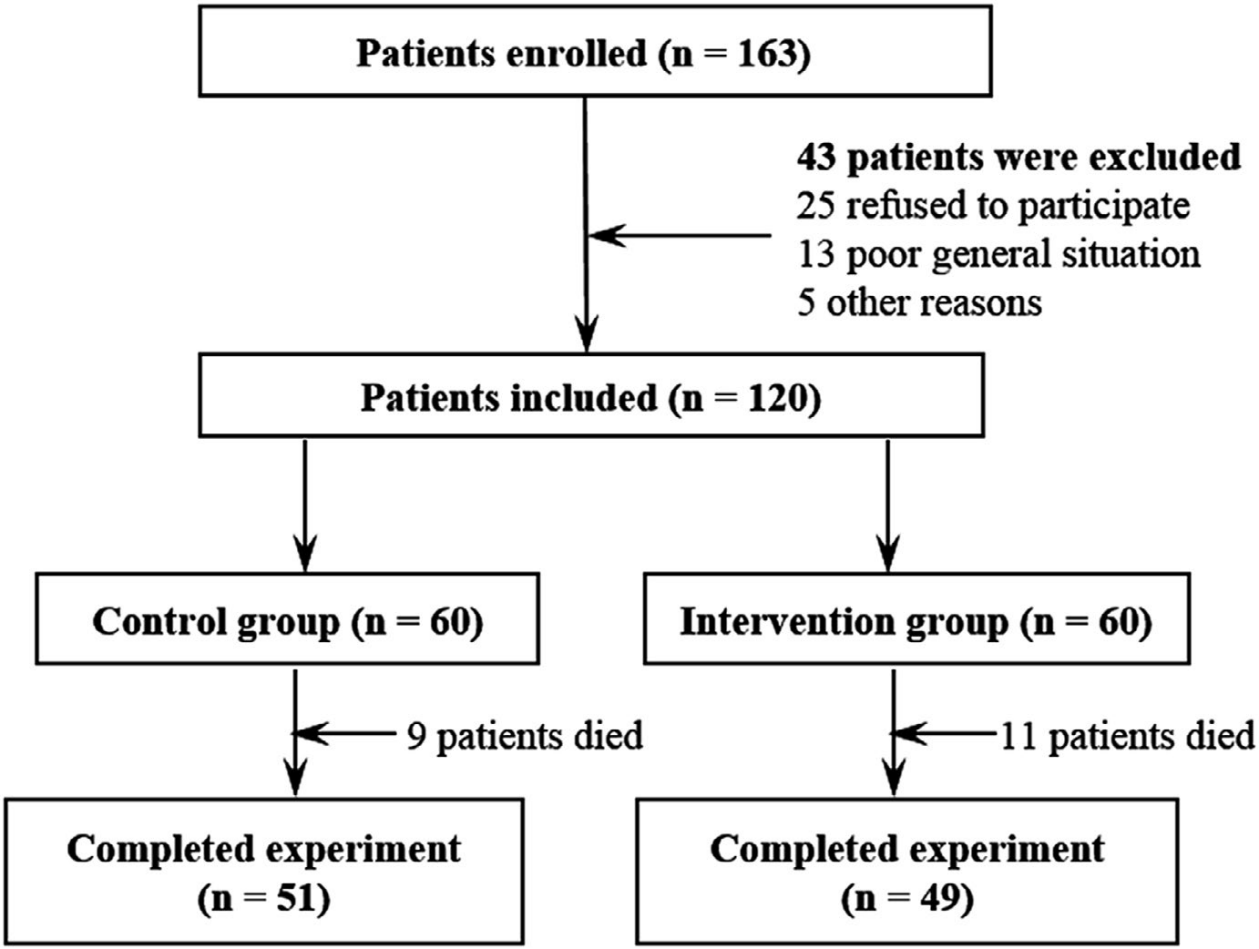
CONSORT flowchart

This prospective randomized controlled clinical trial was approved by the Ethics Committee of the First Affiliated Hospital to China Medical University (No: 2016‐175‐2) and registered at chictr.org.cn with the number of ChiCTR‐IPR‐17011280. All stage III‐IV cancer patients were prospectively enrolled from six research centers from May 10, 2017 to November 10, 2017 (Figure 1).

### 2.2 Inclusion and exclusion criteria

Pathologically or clinically diagnosed stage III‐IV cancer patients were included in the current study according to the following criteria: (1) cancer patients of 18‐year‐old or above; (2) patients with a numeric rating scale (NRS) pain score ≥ 5; Karnofsky performance score (KPS) ≥40; (3) patients with advanced cancer pain cannot take oral analgesics; or take oral morphine at a dose over 100 mg/d without good pain control; or cannot tolerate opioid side effects; and (4) patients voluntarily participated in this study with signed informed consent.

Patients were excluded for the following reasons: (1) refusal to participate in this trial; (2) poor general situation, unable to describe the symptoms; (3) allergy to the medications; (4) inability to tolerate treatment; (5) consciousness disorder; (6) severe infection or respiratory insufficiency; (7) contraindications to test drugs; (8) history of drug abuse; and (9) pregnant, lactating women; or subjects who have a pregnancy plan within 1 month of the trial.

### 2.3 Randomize sequence generation and patient group

According to a computer‐generated random allocation sequence, a total of 120 patients were enrolled and randomly assigned into one of two groups: the intervention group, in which patients received PCSA with sufentainil (n = 60), and a control group, in which patients received PCSA with morphine (n = 60; Figure 1).

**FIGURE 2:**
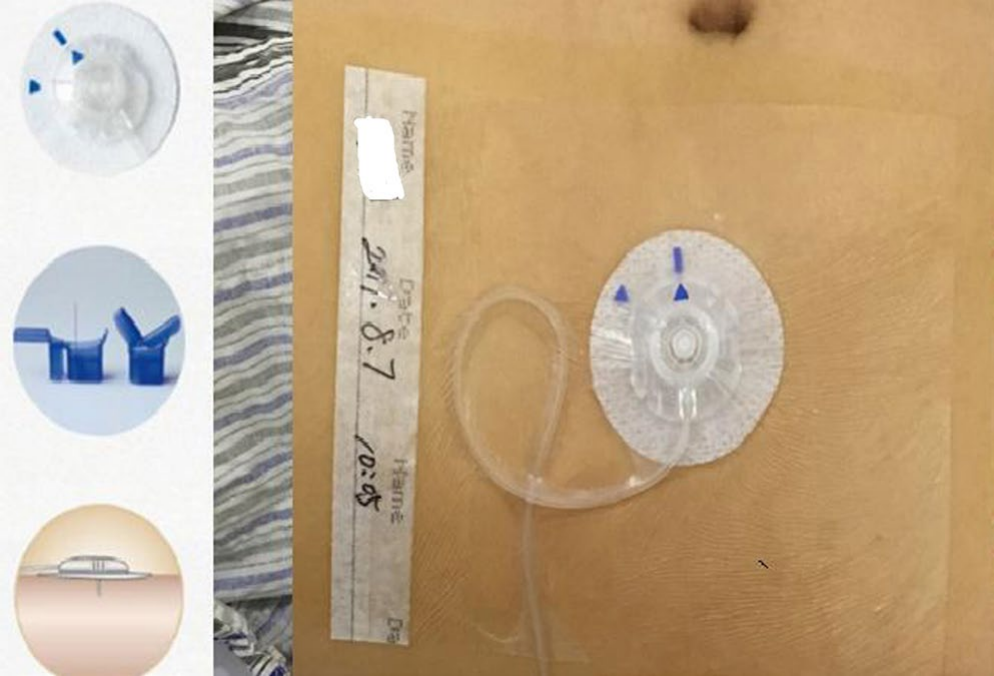
Subcutaneous catheter fixation around the navel

A quick‐set catheter (Medtronic, Northridge, USA) was implanted into the subcutaneous tissue around the navel and the PCA pump was connected ^13^ (Figure 2). In detail, the Tuoren electronic infusion PCA pump (Tuoren Medical, Henan province, China) was used with an integrated bolus‐and‐event recorder. The individual's initial parameters for PCSA, including the drug concentration, the continuous background infusion rate, the bolus dose, the bolus duration, and the lockout time were determined by the baseline dose during the previous 24 hours. Bolus dosing was set at 1 hour after the background opioid infusion with a 30 minute lock out period (the time from the end of one delivery until the device is able to respond to another demand). Patients were instructed to assess the pain level using the NRS before treatment and they could press the bolus button to increase the dose if NRS is over 4.

After randomization, the research associates collected patient information including age, gender, weight, location, and duration of pain. Before the initial treatment (baseline) and on days 1, 3, 7, 14, 28, and 56 post‐treatment, the research associates asked patients to rate their NRS scores. The adverse reactions, including bleeding, infection of the puncture site, subcutaneous induration, nausea, vomiting, urinary retention, and somnolence, were also recorded. All patients were followed up once a week to replace the quick‐set catheter. Additionally, telephone follow‐ups were performed every 3 days to evaluate any adverse reactions. Patients or their family members were able to phone the doctor in case of any emergency.

The initial daily dose of morphine depended on the prior pain treatment. Briefly, the 1/3 dose of oral opioid during the previous 24 hours was given for severe opioid tolerant patients. If the patient suffered from moderate pain (rest NRS score ≧4), a 50% increase in the initial dose was administered.^14^ In our practice, the equivalent dose ratio of sufentanil: morphine was 1:1000.^15^ The program parameters of the PCSA pump were adjusted every 12 hours until sufficient pain relief was achieved (≥50% pain score reduction and break through pain ≤ 3 times a day).

### 2.4 Outcome measures

Data were collected at baseline (1 day before treatment) and in the morning (8:00‐10:00) on days 1, 3, 7, 14, 28, and 56 after the PCSA procedure. The patient data were excluded from analysis if the patient died within the 8‐week follow‐up period.

The NRS scores were evaluated at 8, 16, and 24 o'clock of every measuring day and the average scores were calculated. The Chinese version of the Short Form health survey (SF‐36) ^16^ was used to assess the mental and physical health status of the cancer‐pain patients. The scores of the physical functioning, physical role, body pain, general health perceptions, vitality, social function, emotional role, and mental health index were evaluated at every time point. Side effects, including bleeding and infection of the puncture site, subcutaneous induration, nausea, vomiting, urinary retention, and somnolence/drowsiness, were recorded at the end of the trial.

### 2.5 Treatment of adverse reactions

On the observation of bleeding or infection at the puncture site, the implanted catheter was removed immediately and local antimicrobial treatment was delivered. A new puncture point was located at least 10 cm away from original puncture point. The data of patients with bleeding or infection were collected but not used for the analysis, though they were analyzed based on the intention‐to‐treat principle. A magnesium sulfate solution was used to compress subcutaneous induration. Nausea, vomiting, urinary retention, and somnolence/drowsiness were treated using conventional therapies.

### 2.6 Sample size

According to previous literature, approximately 70% of patients with cancer suffer from moderate to severe pain.^17^, ^18^ We considered a 50% decrease in the pain score to be clinically significant. Thus, we determined that a sample size of at least 48 in each group would enable us to detect a significant difference with a power of 0.8 and a type‐I error of 0.05. We estimated the potential maximal dropout rates as 20%, thus set the final sample size of 120.

### 2.7 Data analysis

The normal distribution of the continuous variables was tested using the Kolmogorov‐Smirnov test and all measurements fell in normal distribution. Values are expressed as mean and 95% confidence interval (CI). Categorical variables were analyzed with Fisher's Exact Test. Statistical analysis was performed using the Statistical Package for Social Sciences version 19.0 (SPSS Inc, Chicago, IL, USA). A *P* value < 0.05 was considered to be statistically significant.

## 3 Results

A total of 163 patients were initially enrolled in this study and 43 patients were excluded for the following reasons: refusal to participate in the study (n = 25), poor general situation (n = 13), and other reasons (n = 5). The demographic characteristics of the patients, including age, gender, body weight, disease duration, and pain score before treatment, were similar between the two groups (Table 1). Nine patients in the control group and 11 patients in the intervention group survived less than 8 weeks, and were analyzed based on the intention‐to‐treat principle (Figure 1).

**TABLE 1:** Baseline patient characteristics

Patients	Control group (n = 51)	Intervention group (n = 49)	*P*
Age (years)	61.9 ± 12.6	65.1 ± 11.8	.627
Female/male, n	26/25	24/25	.778
Weight (kg)	66.9 ± 9.6	66.6 ± 10.4	.832
Disease duration (months)	11.7 ± 3.5	11.1 ± 3.6	.915
Average pain scores	7.4 ± 2.1	7.3 ± 2.3	.887

### 3.1 NRS

**FIGURE 3:**
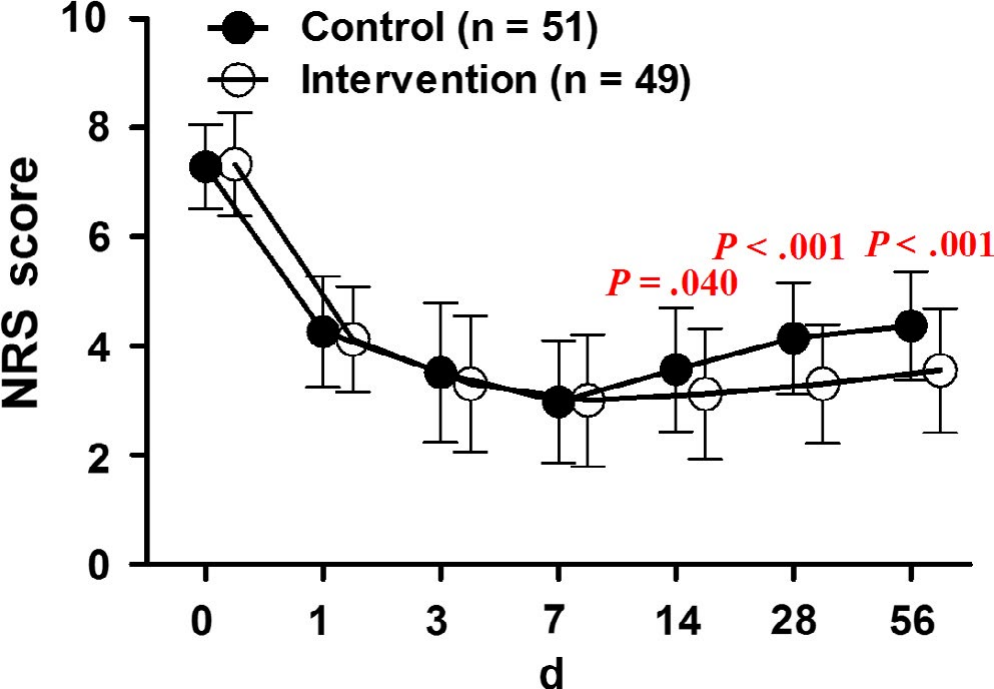
Alterations of the mean numeric rating scale (NRS) scores after patient‐controlled subcutaneous analgesia (PCSA) with morphine (Control) or sufentanil (Intervention). Data were expressed as mean ± SD

There was no significant difference in the mean NRS score between the two groups before treatment (NRS = 7.3, 95% CI: 7.1‐7.5; vs 7.3, 95% CI: 7.1‐7.6 for control and intervention groups, *t* test, *P* = .850). The intervention induced better pain relief indicated by the significantly decreased NRS scores in the intervention group than the control group at 14 (NRS = 3.6, 95% CI: 3.2‐3.9; vs 3.1, 95% CI: 2.8‐3.5 for control and intervention groups, *t* test, *P* = .040), 28 (NRS = 4.1, 95% CI: 3.8‐4.4; vs 3.3, 95% CI: 3.0‐3.6 for control and intervention groups, *t* test, *P* < .001), and 56 (NRS = 4.4, 95% CI: 4.1‐4.7; vs 3.6, 95% CI: 3.2‐3.9 for control and intervention groups, *t* test, *P* < .001) days after pump implantation (Figure 3).

### 3.2 SF‐36

**FIGURE 4:**
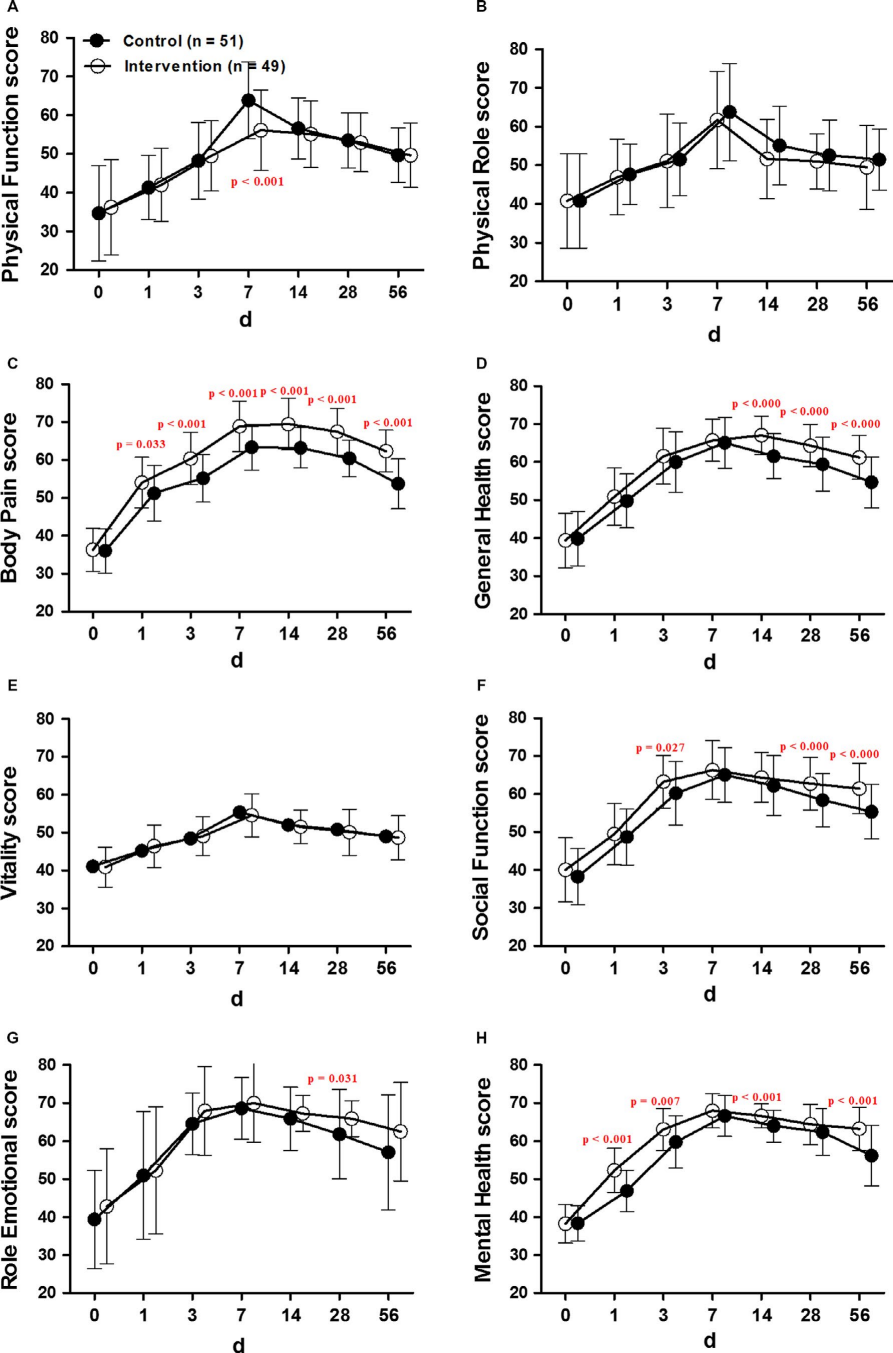
Changes in the SF‐36 parameters after PCSA with morphine (Control) or sufentanil (Intervention). Data were expressed as mean ± SD

There was no significant difference in the baseline SF‐36 scores of varied parameters between the two groups. The intervention induced significant improvement in the SF‐36 parameters except for the physical role (Figure 4B) and vitality (Figure 4E) scores.

#### 3.2.1 Physical function

The intervention induced the significantly increased physical function scores than the control group at 7 (physical function score = 56.1, 95% CI: 53.1‐59.1; vs 63.9, 95% CI: 61.0‐66.7 for control and intervention groups, *t* test, *P* < .001) days after pump implantation (Figure 4A).

#### 3.2.2 Body pain

The intervention induced the significantly increased body pain scores than the control group at 1 (body pain score = 51.2, 95% CI: 49.3‐51.4; vs 54.0, 95% CI: 52.1‐56.0 for control and intervention groups, *t* test, *P* = .033), 3 (body pain score = 55.2, 95% CI: 53.3‐56.9; vs 60.4, 95% CI: 58.5‐62.5 for control and intervention groups, *t* test, *P* < .001), 7 (body pain score = 63.4, 95% CI: 61.0‐66.7; vs 68.9, 95% CI: 53.1‐59.1 for control and intervention groups, *t* test, *P* < .001), 14 (body pain score = 63.2, 95% CI: 61.6‐64.5; vs 69.5, 95% CI: 67.3‐71.3 for control and intervention groups, *t* test, *P* < .001), 28 (body pain score = 60.4, 95% CI: 59.1‐61.9; vs 67.5, 95% CI: 65.6‐69.2 for control and intervention groups, *t* test, *P* < .001), and 56 (body pain score = 53.7, 95% CI: 51.9‐55.7; vs 62.3, 95% CI: 60.6‐63.8 for control and intervention groups, *t* test, *P* < .001) days after pump implantation (Figure 4C).

#### 3.2.3 General health

The intervention induced the significantly increased general health scores than the control group at 14 (general health score = 61.6, 95% CI: 59.9‐63.2; vs 67.1, 95% CI: 65.6‐68.5 for control and intervention groups, *t* test, *P* < .001), 28 (general health score = 59.4, 95% CI: 57.4‐61.5; vs 64.3, 95% CI: 62.7‐66.0 for control and intervention groups, *t* test, *P* < .001), and 56 (general health score = 54.7, 95% CI: 52.7‐56.6; vs 61.2, 95% CI: 59.6‐62.8 for control and intervention groups, *t* test, *P* < .001) days after pump implantation (Figure 4D).

#### 3.2.4 Social function

The intervention induced the significantly increased social function scores than the control group at 3 (social function score = 60.2, 95% CI: 57.8‐62.6; vs 63.3, 95% CI: 61.3‐65.3 for control and intervention groups, *t* test, *P* = .027), 28 (social function score = 58.4, 95% CI: 56.4‐60.4; vs 62.8, 95% CI: 60.7‐64.8 for control and intervention groups, *t* test, *P* < .001), and 56 (social function score = 55.4, 95% CI: 53.3‐57.5; vs 61.5, 95% CI: 59.6‐63.4 for control and intervention groups, *t* test, *P* < .001) days after pump implantation (Figure 4F).

#### 3.2.5 Role emotional

The intervention induced the significantly increased social function scores than the control group at 28 (role emotional score = 61.8, 95% CI: 58.5‐65.2; vs 65.9, 95% CI: 64.6‐67.3 for control and intervention groups, *t* test, *P* = .031) days after pump implantation (Figure 4G).

#### 3.2.6 Mental health

The intervention induced the significantly increased mental health scores than the control group at 1 (mental health score = 46.9, 95% CI: 45.3‐48.5; vs 52.3, 95% CI: 50.7‐54.0 for control and intervention groups, *t* test, *P* < .001), 3 (mental health score = 59.8, 95% CI: 57.8‐61.7; vs 63.1, 95% CI: 61.5‐64.7 for control and intervention groups, *t* test, *P* = .007), 14 (mental health score = 64.0, 95% CI: 62.8‐65.2; vs 66.7, 95% CI: 65.8‐67.6 for control and intervention groups, *t* test, *P* < .001), and 56 (mental health score = 56.2, 95% CI: 53.9‐58.4; vs 63.3, 95% CI: 61.6‐64.9 for control and intervention groups, *t* test, *P* < .001) days after pump implantation (Figure 4H).

#### 3.2.7 Adverse effects

**FIGURE 5:**
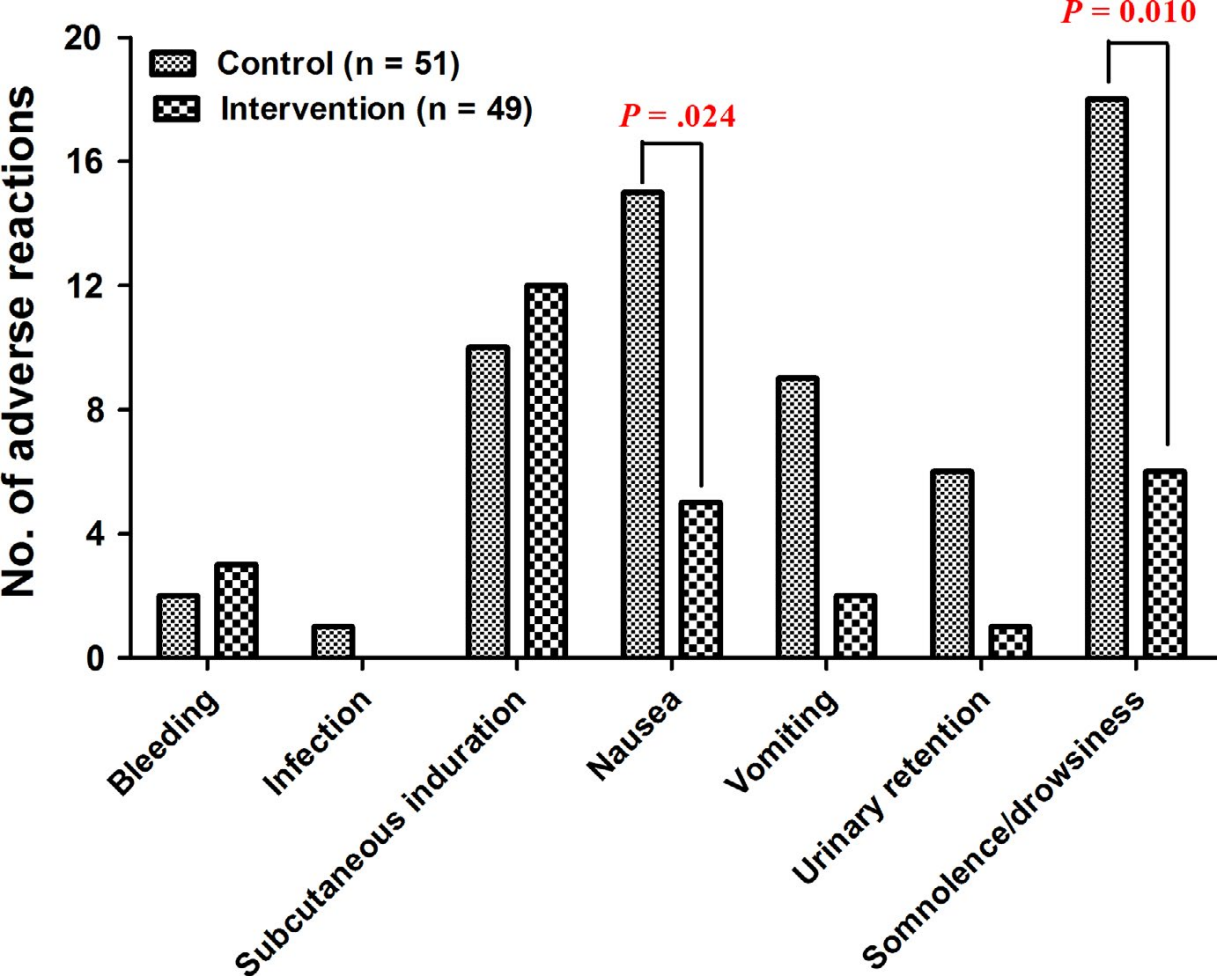
The incidences of adverse events between the two groups. Data were expressed as mean ± SD

The main adverse reactions included bleeding at the puncture site, infection, subcutaneous induration, nausea, and vomiting. Bleeding and local infection at puncture site were mild and recovered quickly after local treatment. However, due to the potential of causing pain by bleeding and infection, the data from these patients were removed from analysis and the patients were analyzed based on the intention‐to‐treat principle. There was no significant difference in the observed adverse reaction rates between the two groups (*P* > .05) except for the nausea (Fisher's exact test, *P* = .024) and somnolence (Fisher's exact test, *P* = .010; Figure 5).

## 4 DISCUSSION

PCSA with morphine or sufentainil can effectively relieve pain in patients with stage III‐IV cancer. Sufentainil provides better analgesia, improved life quality, and lower levels of nausea and somnolence events than PCSA with morphine. No significant difference was observed in other adverse reactions between the two groups.

There are approximately 4 million patients diagnosed with malignant tumors each year in China^19^ and 60%‐70% of these patients will develop cancer pain at terminal stage.^20^ The vast majority of these patients could obtain satisfying analgesia with sustained release of morphine tablets and other non‐invasive medications following the WHO analgesic ladder.^21^ However, when oral delivery of medication is difficult, pain control is poor and remains a clinical challenge. Furthermore, sustained‐release morphine tablets are not effective in controlling bursts of breakthrough pain. Another issue that needs to be addressed is that patients with stage III‐IV cancer have a variety of symptoms and are often suffering from pain, resulting in a low quality of life.^22^ Thus, a better alternative analgesia with improved life quality is required for these patients.

Patient‐controlled intravenous analgesia (PCIA) and PCSA are two common techniques that are used to maintain drug concentrations to be stable enough to meet the analgesia requirement of patients. Terminal cancer patients in China cannot access required medical resources due to the limited hospital resources and severe financial burden. We aimed to obtain better and safer pain relief for patients with stage III‐IV cancer. In agreement with previous reports, in the current study, PCSA sufentanil provided comparable or improved rapid onset analgesia compared to that with morphine. Meanwhile, PCSA sufentanil provided a better life quality and fewer adverse events than that with morphine, which is consistent with a small population pilot study in 1995.^11^

No severe adverse event was observed in the current study. The likely reasons are as the follows: (1) PCSA is a safe drug delivery technique, as reported in a previous study.^11^ (2) Regular reexamination and telephone follow‐ups may decrease the risk of damaging adverse events.

This study has limitations. First, this study was performed in only one province and further multi‐site investigations are necessary to validate the conclusion. Second, it was difficult to recruit a large number of patients because 26.4% of the eligible patients refused to participate, due to their unwillingness to undergo PCSA, which may have led to the selection bias.

## 5 CONCLUSION

PCSA with opioid or sufentainil in home care could effectively relieve stage III‐IV cancer pain. Sufentainil PCSA could provide a better analgesia, improved life quality, and less adverse events than morphine.

## CONFLICT OF INTEREST

The authors declare that they have no conflict of interests.

## AUTHOR CONTRIBUTION

Cheng‐Fu Wan: clinical experimental design, clinical trial practice; statistical analysis of data and manuscript writing; Qing‐Zhu Meng: clinical trial practice; Yan‐Wei Wang: clinical trial practice; Liang Qi: clinical trial practice; Chang‐Liang Ai: clinical trial practice; Xin Sui: clinical trial practice; Tao Song: clinical experimental design and manuscript review.

## ETHICAL APPROVAL

This prospective randomized controlled clinical trial was approved by the Ethics Committee of the First Affiliated Hospital to China Medical University (No: 2016‐175‐2) and registered at chictr.org.cn with the number of ChiCTR‐IPR‐17011280. All procedures performed in studies involving human participants were in accordance with the ethics standards of the institutional and national research committee and with the 1964 Helsinki Declaration and its later amendments or comparable ethics standards.

## Data Availability

The data used and/or analyzed during the current study are available from the corresponding author on reasonable request.
